# The Marquis of Salisbury at Oxford

**Published:** 1893-03-11

**Authors:** 


### The Hospital

An Institutional Journal of

Science, Medicine, Nursing, and Philanthropy.

*For Hospitals, Asylums, Infirmaries, Dispensaries, Medical Practitioners, Students, Nurses, and the Charitable Public.*

Vol. XIII.—No. 337.] [March. 11, 1893.

### THE MARQUIS OF SALISBURY AT OXFORD.

In his speech on behalf of the Extension Fund of the Radcliffe Infirmary last week, the Marquis of Salisbury quoted a sentence from what he called "a friendly critic," and that friendly critic was the *Hospital Annual.* It is the fact that The Hospital and the *Hospital Annual* have had a controversy with the authorities of the Radcliffe Infirmary, which has extended over nearly two years. On a considerable number of occasions it has been charged against The Hospital, as it was at Oxford, that it is a fault-finding organ, not controlled by that profound sense of responsibility which is the most marked characteristic of the worthiest English journalism. This account of ourselves we resolutely challenge, and we point our contention by a brief history of what has happened at Oxford since the date of April 11th, 1891.

On April 11th, 1891, there appeared in the columns of The Hospital an article written by our "Special Commissioner" on the "Radcliffe Infirmary, Oxford." Our Commissioner had visited the infirmary for a particular purpose; but an inspection of the building revealed to him circumstances of such moment, that he practically forgot the lesser purpose for which he had originally journeyed to the city of towers, and set himself upon the much more serious task of opening the eyes of the local authorities to the inefficient and unworthy condition of their city infirmary when compared with the general hospital standards of the day. Perhaps it is superfluous to say that our Commissioner did not mince matters. What, indeed, is the use of mincing matters when one is dealing with public questions of serious and urgent import? Science always calls "a spade a spade," and people of education for the most part set upon such truthfulness and simplicity a very high value. Here is a sample of our Commissioner's "plainness of speech!" "The Radcliffe Infirmary is entered by the basement. The entrance is situated in the oldest part of the buildings, which present an altogether neglected and miserable appearance. The fittings and appliances are for the most part of the oldest and worst description. . . . We venture to say that it would be difficult to find a parallel to the present condition of many parts of the Radcliffe Infirmary amongst the voluntary institutions of this country. . . . The condition of the wards so far as the walls, the sanitary fittings, baths, and other appliances are concerned, exhibits an almost total disregard not only for appearances, but also for sanitary requirements. . . . We can honestly say that in the course of an experience extending over a quarter of a century we never remember to have visited a hospital with a feeling of greater interest, or to have left it with a feeling of greater depression and astonishment."

Not unnaturally the authorities of the Radcliffe Infirmary read those charges with extreme surprise, a surprise which deepened into anger. They could not believe that the facts were so bad as they were stated to be, although they had been looking upon those very facts for years. To believe that such charges were true was to believe things about themselves of so unworthy a kind that all the native self-respect of the well-intentioned Briton rose up in arms against the traducer. Familiarity had, in fact, produced blindness, if not hardness of heart. The authorities protested; they protested too much, and in the very act of protesting they gave away their own case: "Vowing they would ne'er consent, they consented." Within a month of the publication of our Commissioner's article Dr. Bright, Master of University College, and Treasurer of the Infirmary, made an appeal to the public for £10,000 to carry out the very suggestions for the improvement of the wards and buildings which our Commissioner had made.

Then came another phase of the story. The authorities were willing, but the public were not. The appeals made for subscriptions produced practically no response. Here, again, was the opportunity of The Hospital. It had severely criticised the authorities when they were undoubtedly to be blamed. Now it came to their aid. We do not wish to take any credit to ourselves at all for this: we only desire to make it plain to the managers at Oxford and to other managers whom we may have assailed or may yet feel bound to assail elsewhere, that we acted openly and righteously upon the actual facts of the case.

Had our Commissioner done other than he did he would have been recreant to his plainest duty, and unworthy of the proud and honest name of "John Bull." The truth is that finesse is out of date in public affairs. Misrepresentation, petty criticism for the sake of notoriety, vulgar sensationalism we hate with all our hearts. But all the more is it incumbent upon us to try to rise to that highest effort of public service to which any journalism can attain, and to speak the truth without fear or favour, in the highest interests of all.

Lord Salisbury did us the honour and the justice of calling our criticism "friendly." May we be pardoned if we venture a little further into the sensitive region of friendly criticism? It is evident now that the authorities of the Radcliffe Infirmary will obtain the money they require, and probably more. What will they do with it? Ten thousand pounds is a considerable sum, and can effect great objects if competently spent. What are the authorities going to do for the efficient sanitation of the old parts of the building? Above all, where will they place the new? Former additions have shown a disposition, like Dean Ramsay's ghost, to "take a meander through the hospital garden." But that will not—must not—be repeated. What is obviously the solution of the difficulty is a circular ward, and a circular ward placed at the junction of the present wings, where light and air are ample, and where an efficient administrative connection with the main building can be obtained with the minimum of difficulty.

Our constant plea with Oxford has been that she shall make her work worthy of herself. It is, of course, the highest claim we can make upon any man or city. But if such a claim may not be made upon Oxford, the home of literature, the home of culture, the home of religion, where may it be made? Here is a great work to be done. It ought to be so done that the most critical of critics who beholds it completed shall be compelled to say, "It is done well." A mean outside must be carefully avoided, and inadequate internal appliances must be held to be impossible. It will not in future be the eyes of The Hospital Commissioner which will be fixed upon Oxford and her Radcliffe Infirmary, but the eyes of the medical profession, of all sanitarians, of the whole British public indeed, to whom Lord Salisbury's speeches are as oracles and as words to be treasured for all time.

If it be wondered why we should be so desirous of seeing Oxford's Infirmary so absolutely perfect both within and without, our reply is that we stand for hospitals and for medicine, and we must stand for the absolute best. Medical men are bound by every imaginable consideration to aim at the best. It would be shocking that they should do otherwise. Everything connected with medicine should naturally, and as a matter which admits of no question, be of the very highest which can be attained to; the highest personal skill and culture, the best remedies, the best hospitals, the best appliances. Does not this contention at once commend itself to the mind of every man? Anything short of the best in medicine is a contradiction and an absurdity. Relative medicine would be like relative morals, or a relative wife, inconceivable as a standard and an aspiration. Once more, and with full confidence, we appeal to the culture and to the faith of Oxford to give the city and the county an infirmary which for practical efficiency in all the requirements of an ideal hospital shall be second to none in the world.

